# Are Japanese spousal terms as gender-biased as they seem? An examination using implicit association measures

**DOI:** 10.1371/journal.pone.0330816

**Published:** 2026-06-03

**Authors:** Ri Nin, Kazuo Mori

**Affiliations:** Institute of Engineering, Tokyo University of Agriculture and Technology, Koganei, Tokyo, Japan; McGill University Faculty of Arts, CANADA

## Abstract

This study investigated whether traditional Japanese spousal terms—*shujin* (“master”) and *kanai* (“inside-the-house”)—retain male-dominant associations at an implicit level. Using a paper-based Implicit Association Test (the FUMIE Test), we compared implicit attitudes toward traditional and neutral spousal terms among Japanese university students. The results showed no significant difference between the two term pairs, suggesting that *shujin* and *kanai* may have become conventionalized rather than continuing to evoke their hierarchical literal meanings. However, a significant gender difference emerged: male participants evaluated male-associated terms more positively, whereas female participants showed no such bias. These findings indicate that while traditional terms may no longer strongly activate hierarchical associations at an implicit level, latent gender asymmetries persist, particularly among men. The study highlights the importance of examining implicit dimensions of language use and provides a useful methodological tool for investigating gender bias in sociolinguistic research.

## Introduction

Gender equality remains an ongoing challenge in East Asia, where traditional patriarchal values—such as the notion of male superiority (男尊女卑*dan-son jo-hi*) and the ideal of a wife following her husband (夫唱婦随*fu-sho fu-zui*)—continue to shape social norms [1,2]. Linguistic expressions, especially spousal terms, serve as reflections of such values. Prior studies have shown that spousal terms in Japan, China, and South Korea often encode gendered power dynamics, with Japanese terms in particular exhibiting strong male-dominant connotations [3–5].

According to recent surveys by Nin, the most commonly used Japanese spousal terms are *shujin* (主人; master/husband) and *kanai* (家内; inside-the-house/wife). The term for the male spouse consists of two Chinese characters: “主” meaning “main” or “primary,” and “人” meaning “person,” together conveying the idea of a “master.” In contrast, the female term comprises “家” (house) and “内” (inside), denoting someone confined within the home. These terms encapsulate the traditional male-centric family model in Japan, in which the husband is the master of the household and the wife belongs inside it. In comparison, the Chinese equivalents are *laogong* (老公; old lord) for husbands and *laopo* (老婆; old granny) for wives [3]. Nin’s expanded survey found that Korean spousal terms—*ba-kkat-yang-ban* (바깥양반; outer nobleman) and *jib-sa-lam* (집사람; person at home)—closely resemble the Japanese system [5], while in Indonesia, traditional terms like *mas/aa/akang/kak* (big brother) and *dik* (little one) have largely been replaced by the more neutral *suami* (husband) and *istri* (wife) [6].

Spousal terms vary across languages, cultures, and historical periods, reflecting broader social ideologies. In Japanese, terms such as *shujin* and *kanai* have long been criticized for perpetuating hierarchical marital roles. Despite these criticisms, they are still widely used today, as shown by national surveys [7], linguistic analyses [8–13], and recent studies by Nin [4].

What is especially noteworthy is that Japan—despite its early and extensive Westernization—displays a greater degree of gender disparity in spousal terms than countries like China and Indonesia. This finding challenges the commonly held belief that modernization and Westernization automatically promote gender equality [14]. The pattern may be attributed in part to political and ideological shifts in post-revolutionary China. Such observations highlight the complexity of gendered language and the limitations of Westernization as a sole driver of cultural change.

Nin [4] also found that the more neutral spousal terms *otto–tsuma* (夫–妻; husband–wife) were the second most frequently used after the traditional pair *shujin*–*kanai* (主人–家内; master–inside-the-house). Notably, while *otto* (夫; husband) was the second-most preferred term among men, *tsuma* (妻; wife) was the top choice among women, far surpassing the traditional counterpart *kanai* (家内; inside-the-house). The study also revealed that the higher the respondent’s educational level, the more likely they were to use the neutral terms.

Despite growing scholarly interest in gender and language, most previous studies have focused on explicit usage without exploring the psychological mechanisms that sustain these patterns. To address this gap, the present study integrates linguistic analysis with a psychological technique known as the Implicit Association Test (IAT; [15]). The IAT enables researchers to assess unconscious associations by measuring response times in categorization tasks. If a term is more strongly associated with positive concepts, it will be categorized more quickly when paired with other positive stimuli than with negative ones. This method allows researchers to detect subtle, implicit biases that may not be revealed through self-report data.

In this study, a paper-and-pencil IAT—the FUMIE test [16]—was used to examine Japanese university students’ implicit attitudes toward two sets of spousal terms: the traditional pair *shujin*–*kanai* (主人–家内; master–inside-the-house) and the neutral pair *otto*–*tsuma* (夫–妻; husband–wife). Participants were asked to associate these terms with either positive or negative concepts, and their reaction speeds were measured. If no significant differences are found between the two term pairs, it may indicate that the traditional spousal terms no longer strongly activate their original hierarchical connotations and are now used in a neutral, conventional manner, similar to the neutral counterparts. However, if the traditional terms elicit stronger positive or negative associations than the neutral ones, it would suggest the persistence of latent gender bias of the traditional spousal terms.

Accordingly, this study addresses the following key research question:

Are male-dominant terms such as *shujin* (主人; master) and *kanai* (家内; inside-the-house) in Japanese merely symbolic, or do they continue to reflect hierarchical gender attitudes at an implicit (unconscious) level?

## Method

### Ethics statement

The study was approved by the Institutional Review Board of Tokyo University of Agriculture and Technology in 2025 (Approval ID: 250805−0740). All data were collected and analyzed anonymously.

### Participants

A total of 162 male and 84 female undergraduate students majoring in engineering and agriculture participated in the study after providing informed consent. Participants were fully informed about the purpose of the study, participation was entirely voluntary, and all data were collected anonymously. Participants were also informed that submission of the completed test sheet would indicate their consent to participate.

### Construction and logic of the FUMIE test

The implicit connotations of two pairs of spousal terms were assessed using the FUMIE Test [16], a paper-based task designed to measure implicit associations through speeded word evaluation. The A3-sized test sheet included rows of words with either positive or negative connotations (hereafter referred to as “evaluative words”). The two target pairs—*shujin* (“master”) vs. *kanai* (“inside-the-house”), and *otto* (“husband”) vs. *tsuma* (“wife”)—were embedded at regular intervals in a pseudo-random order among the evaluative items. Each line contained a balanced and randomized mix of evaluative words and target terms, arranged to prevent the consecutive appearance of target words.

To clarify the role of the control condition, the neutral spousal terms, *otto* (“husband”) vs. *tsuma* (“wife”), were included as a baseline against which the implicit associations of the traditional terms, *shujin* (“master”) vs. *kanai* (“inside-the-house”), were evaluated. The neutral terms were selected to be semantically non-hierarchical and emotionally neutral, while remaining comparable to the traditional terms in lexical category and usage frequency. Apart from the semantic content of the target words, all task parameters (stimulus format, number of items per line, presentation order, time limits, and scoring procedures) were identical across the traditional and neutral conditions. Thus, any observed differences in performance are likely attributable primarily to differences in the semantic associations elicited by the spousal terms rather than to procedural factors.

For this study, a 16-line FUMIE Test sheet was created. The first 7 lines were used to assess the traditional spousal pair, and the next 7 lines for the neutral pair, with the remaining 2 lines serving as dummy trials to prevent end-of-test speeding (“final-effort” bias) near the 14th line. In each section, the first line served as a practice trial and did not include any target words. From the second line onward, target terms were embedded and used for assessment. A sample FUMIE test sheet used in the present study is provided as Supporting Information (S1 Appendix).

Participants were instructed to mark each target word with either a circle or a cross, regardless of their actual attitudes—placing a circle on *shujin* and a cross on *kanai*. (This circle-and-cross response format is a familiar and widely used convention in Japan.) Furthermore, the combination of markings was reversed every other line (i.e., alternating combinations), with each condition repeated across three lines.

The FUMIE Test is based on the principle that individuals complete tasks more quickly when the required response (e.g., marking a circle) aligns with their implicit attitudes. Thus, a participant with a positive implicit attitude toward a target word will respond more quickly when instructed to mark that word with a circle than with a cross, and vice versa. The difference in performance between the two task conditions (positive vs. negative combinations) serves as an index of the participant’s implicit attitude toward the term pair. Specifically, a participant with traditional gender attitudes would respond more quickly in the positive combination condition (circle on “master,” cross on “inside-the-house”) than in the negative combination (the reverse).

The present study was particularly interested in examining a second-order effect, namely whether the performance difference between the two task combinations differs depending on the spousal term pair. If the magnitude of difference between positive and negative combinations is greater for the traditional pair (*shujin–kanai*) than for the neutral pair (*otto–tsuma*), it would suggest that the traditional terms preserve stronger hierarchical or male-centric connotations. Conversely, if the differences are comparable across both term pairs, it would imply that their implicit connotations are similarly neutral.

### Administration procedure of the FUMIE test

The FUMIE Test was administered by one of the authors during undergraduate classes on 8 April 2025 (Tuesday) and 10 April 2025 (Thursday). As described above, the assessments using the two spousal term pairs were conducted successively on a single test sheet without interruption between the two conditions.

The instructor distributed the test sheets and provided instructions using a set of PowerPoint slides. Before the assessment began, participants were assured that their responses would remain anonymous and were asked to indicate their gender and age at the top of the sheet.

General instructions were then given in accordance with the *FUMIE Test Administration Manual* (Ver. 2.2; [17]). For the first line, participants were instructed to classify each word as either “good” or “bad” by marking a circle for the former and a cross for the latter, as quickly as possible within 20 seconds. This served as an orienting task and a practice trial.

From the second to the seventh lines, the target word pair *shujin* (“master”) and *kanai* (“inside-the-house”) appeared in a pseudo-random order among the evaluative words. Participants were instructed to mark a circle for *shujin* and a cross for *kanai* on the second, fourth, and sixth lines, regardless of their own judgment, and to reverse the markings (i.e., a cross for *shujin* and a circle for *kanai*) on the third, fifth, and seventh lines. Each line was to be completed within 20 seconds. The same procedure was repeated for the eighth through the 14th lines using the other spousal term pair, *otto* (“husband”) and *tsuma* (“wife”).

Finally, the instructor reminded participants to confirm their age and gender information and emphasized again that submitting the completed test sheet constituted voluntary participation. The test sheets were then collected, and the instructor thanked the students for their cooperation. The entire procedure took approximately 10 minutes.

## Results

All students agreed to participate in the study and submitted their test sheets. The total sample included 246 students: 162 male students (aged 18–24, *M* = 19.6) and 84 female students (aged 18–24, *M* = 19.2). Among them, 186 were majoring in engineering and 60 in agriculture; however, academic major was not considered in the following analyses.

First, for each participant, we counted the number of words marked under the positive combination (WP; i.e., marking “master/husband” as “good” and “inside-the-house/wife” as “bad”) and the negative combination (WN; i.e., marking “master/husband” as “bad” and “inside-the-house/wife” as “good”). We then calculated the Implicit Association Quotient (IAQ_100_) using the following formula:


$$\begin{array}{c} {𝐈𝐀𝐐}_{100}=100\times (𝐖𝐏-𝐖𝐍)/(𝐖𝐏+𝐖𝐍). \end{array}$$

The IAQ_100_ represents the normalized difference in performance between the positive and negative combinations, scaled to a 100-point metric. A positive IAQ_100_ score indicates that the male spousal words were implicitly associated more positively than the female spousal words, while a negative score suggests the opposite direction.

Test examinees may occasionally fail to respond appropriately due to momentary lapses of attention or other accidental reasons. Although such erroneous responses can be excluded on a response-by-response basis in the IAT procedure, no standard procedure exists for handling erroneous responses in the performance-based FUMIE test. Nevertheless, responses showing clear signs of interruption or excessive variability should be excluded to avoid contaminating the results. The *FUMIE Test Administration Manual* (Ver. 2.2; [17]) recommends excluding data on a case-by-case basis according to the statistical error distribution, treating values deviating more than ±1.96 standard deviations from the mean as outliers, which fall outside the range expected for 95% of observations under random error. Accordingly, data from 16 male and 6 female students were excluded because their scores exceeded ±2 standard deviations. Subsequent analyses were conducted using the remaining 224 participants (146 male and 78 female students).

**Fig 1:**
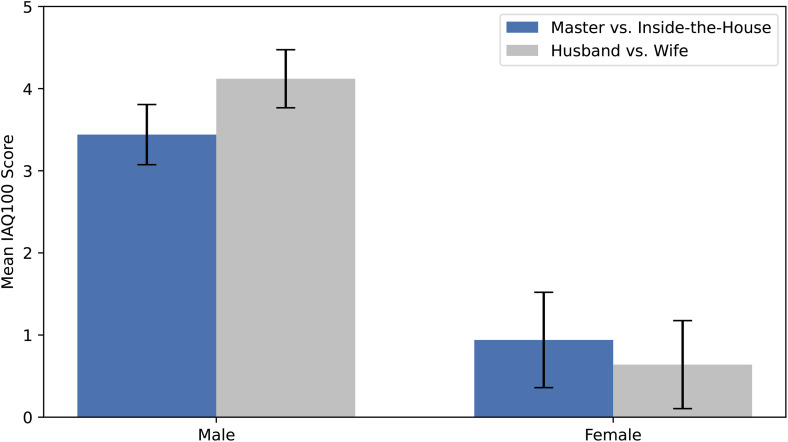
Mean IAQ_100_ scores for traditional (Master vs. Inside-the-House) and neutral (Husband vs. Wife) spousal terms, separated by participant gender. Error bars represent standard errors.

The mean IAQ_100_ scores by participant gender for the two spousal term pairs are shown in Fig 1. On average, male participants implicitly evaluated both spousal term pairs more favorably toward male-associated terms. In contrast, female participants showed no particular preference in either direction.

A two-way mixed-design ANOVA was conducted on the IAQ_100_ scores, with gender (male vs. female) as a between-subjects factor and spousal term (master/inside-the-house vs. husband/wife) as a within-subjects factor. The analysis revealed a statistically significant main effect of gender, *F*_(1,222)_ = 39.94, *p* < .00001, partial *η*² = .1525, indicating that male and female participants differed in their IAQ_100_ scores. However, the main effect of spousal term pair was not statistically significant, *F*_(1,222)_ =.18, *p* = .672. The interaction between gender and spousal term was also not significant, *F*_(1,222)_ = 1.28, *p* = .259.

The observed effect size for the main effect of spousal term was small (partial *η*² = .0057, Cohen’s *f* = .076). An a priori power analysis indicated that the present sample size (*N* = 224) provided more than 99% power to detect a large effect (*f* = .40) at *α* = .05. The choice of *f* = .40 as the criterion for a large effect follows Cohen’s conventional benchmark and is further justified by the fact that the gender effect observed in the same dataset yielded a comparably large effect size (*f* = .4241). Furthermore, the 95% confidence interval for the effect size yielded an upper bound of approximately *f* = .207, indicating that effects of large magnitude (*f* ≥ .40) can be statistically ruled out. Taken together, these results suggest that the absence of a significant difference between the two Japanese spousal terms in the present study is unlikely to be attributable to insufficient statistical power and instead supports the conclusion that no practically large difference exists between them in this sample.

These results indicate that the two sets of spousal terms were implicitly evaluated in a similar manner by both male and female participants, as no significant difference was observed in IAQ_100_ scores across the term pairs. In contrast, the significant main effect of gender—with a large effect size (partial *η*² = .1525, *f* = .4241)—indicates that male and female participants differed substantially in their overall implicit evaluations. Male participants showed a clear tendency to evaluate male-associated terms more positively, whereas female participants showed no strong directional bias. Although the mean IAQ_100_ scores of female participants were slightly positive, they fell within the margin of error around zero as defined in the FUMIE manual [17].

## Discussion

### Overview of the research purposes and findings

The central research question of the present study was whether the traditional spousal terms *shujin* (主人; “master”) and *kanai* (家内; “inside-the-house”) are merely symbolic or whether they continue to reflect male-dominant associations or attitudes at an implicit level. To address this question, we examined the implicit associations of Japanese spousal terms using a paper-based Implicit Association Test (the FUMIE Test), focusing on two term pairs: the traditional *shujin–kanai* (“master–inside-the-house”) and the neutral *otto–tsuma* (“husband–wife”).

The results revealed comparable IAQ_100_ score patterns across the two spousal term pairs for male and female participants. However, a significant gender difference emerged in the IAQ_100_ scores: male participants implicitly evaluated both pairs more favorably toward male-associated terms, whereas female participants showed no strong directional bias.

### No difference found between traditional and neutral spousal terms

Nin’s prior studies [3–5] found that Japanese spousal terms exhibit stronger male-dominant connotations than those in Chinese and Korean, although the three countries share broadly similar East Asian cultural backgrounds. The spousal terms most commonly used in Japan are *shujin* (主人; “master”) and *kanai* (家内; “inside-the-house”), which literally encode a traditional gendered division of roles. These patterns have raised the question of why Japan appears to lag behind China in reducing gender disparity in spousal-term usage, despite its earlier and more extensive Westernization.

The present study addressed this issue using a somewhat unique approach by assessing the implicit association patterns of the traditionally “male-dominant” spousal terms (*shujin–kanai*) in comparison with more neutral terms (*otto–tsuma*). Crucially, no significant difference was observed between the two spousal term pairs. Importantly, this null effect was not attributable to insufficient statistical power, and effects comparable in magnitude to the observed gender difference can be confidently ruled out. This suggests that, unlike gender, Japanese spousal terms may not elicit practically large differences in implicit responses in the present population.

These findings imply that the traditional pair *shujin–kanai*, although often regarded as male-dominant in explicit discourse, may not be evaluated more negatively than the neutral *otto–tsuma* pair at an implicit level—at least among university students. One possible explanation is that these traditional terms have become lexicalized and are used as conventional labels without strong activation of their original hierarchical meanings.

### Gender differences reflected in Japanese spousal terms

Although the findings of this study suggest that *shujin* and *kanai* are not necessarily perceived as strongly male-centric at an implicit level, they nevertheless indicate that latent gender-related asymmetries remain in how Japanese spousal terms are implicitly evaluated. Specifically, a statistically significant gender difference emerged in IAQ_100_ scores: male participants implicitly evaluated male-associated terms more positively than female-associated terms, whereas female participants showed no strong directional bias.

This asymmetry between male and female responses suggests that implicit evaluations of gendered language may differ systematically by gender within this sample. A more gender-symmetric pattern would be characterized by comparable implicit responses across male and female participants. However, the present results deviated from such symmetry, with male participants showing a clear positive bias toward male-associated terms, whereas female participants remained approximately neutral.

In other words, both traditional and neutral spousal terms elicited a similar pattern among male students, with male-associated terms being evaluated more positively, whereas female students appeared to evaluate male- and female-associated terms more evenly. This pattern may reflect a tendency toward greater implicit neutrality among female participants in this cohort, whereas such neutrality was not observed among male participants. Given the age and educational background of the sample, these findings highlight ongoing gender-related asymmetries in implicit language evaluations among Japanese university students, rather than allowing strong generalizations about broader societal trends.

In addition, the present study implicitly treated dominance-related meanings as aligned with evaluative valence. However, dominance and valence are conceptually distinct dimensions, and dominance-related concepts are not necessarily evaluated as positive or negative. Therefore, the interpretation of implicit associations in terms of hierarchical meaning should be made with caution. Further research is needed to examine dominance-related semantic dimensions independently from affective valence.

### Limitations and future directions

This study addresses the question of why Japanese spousal terms appear more male-centric than their counterparts in China or Korea by examining their implicit associations. The findings suggest that these traditional Japanese terms may not strongly activate male-dominant associations at an implicit level, even though they are often perceived as male-dominant in explicit discourse. Furthermore, the study demonstrates the utility of a paper-based Implicit Association Test (IAT) for examining gendered language use by assessing speakers’ implicit cognitive structures. This approach provides a practical methodological contribution that may be applicable to research on gender and language across different linguistic and cultural contexts.

A conceptual limitation of the present study concerns the distinction between semantic properties of spousal terms and more general gender-based evaluations. Because spousal terms are inherently gender-marked, the implicit associations captured by the FUMIE test may reflect not only evaluative meanings embedded in the terms themselves but also broader gender stereotypes associated with men and women. The current design does not allow these two sources of bias to be fully disentangled. Future studies could address this issue by including additional control terms or by orthogonally manipulating gender marking and semantic content.

In addition, several further limitations should be acknowledged. First, the sample consisted exclusively of university students from science-related fields, which may limit the generalizability of the findings. Second, the study focused solely on Japanese spousal terms; cross-linguistic comparisons with Chinese, Korean, or Taiwanese terms—as suggested by Nin [5]—remain to be explored. Third, although the FUMIE Test captures implicit associations effectively, it does not address long-term language change or the pragmatic use of such terms in naturalistic discourse.

Future research should expand the participant pool to include individuals from more diverse age groups, educational backgrounds, and sociocultural contexts. It should also further investigate how implicit gender-related language evaluations evolve over time and across cultural settings. Such efforts will contribute to a more comprehensive understanding of how traditional values persist or transform in everyday language use.

## Conclusions

This study examined whether traditional Japanese spousal terms—*shujin* (“master”) and *kanai* (“inside-the-house”)—retain male-dominant associations at an implicit level. Using a paper-based Implicit Association Test (the FUMIE Test), we compared participants’ implicit evaluations of traditional and neutral spousal term pairs.

In contrast to prior sociolinguistic descriptions emphasizing the hierarchical meanings of these terms, the results revealed no significant difference in implicit evaluations between the traditional (*shujin–kanai*) and neutral (*otto–tsuma*) pairs. This pattern suggests that, among university students, the traditional terms may have become conventionalized and may no longer strongly activate hierarchical associations at an implicit level.

However, a significant gender difference emerged. Male participants evaluated male-associated terms more positively, whereas female participants showed no strong directional bias, indicating that latent gender-related asymmetries persist, particularly among men. These findings underscore the importance of considering both explicit and implicit dimensions when evaluating gendered language use.

While the study demonstrates the utility of a paper-based IAT approach for examining implicit language attitudes, it also highlights the need for further cross-cultural and generational research. Accordingly, the present findings should not be interpreted as direct evidence about the evaluative semantics of specific spousal terms per se, but rather as reflecting implicit associative patterns that may combine lexical meanings and more general gender-related evaluations.

## Supporting information

S1 Appendix FUMIE Test Sheet English Sample. In this English sample, Japanese stimuli have been replaced with English words to illustrate the overall format of the FUMIE test. In the actual test sheet, however, all stimuli are presented as two-character Japanese kanji compounds, allowing words—including the embedded target terms (*shujin*, *kanai*, *otto*, and *tsuma*)—to be arranged at equal intervals within each row. Because Japanese kanji compounds require less space, 60 words can be printed in each row in the original Japanese version. Correspondences between the original Japanese stimuli and their English equivalents are provided in a separate sheet. Participants are instructed to judge, as quickly as possible within 20 seconds, whether each word has a positive or negative meaning, and to mark each word with a circle (○) or a cross (×).
(XLSX)
